# Numerical simulations of tunable ultrashort power splitters based on slotted multimode interference couplers

**DOI:** 10.1038/s41598-019-49186-x

**Published:** 2019-09-04

**Authors:** Chia-Chien Huang, Te-Chia Sun

**Affiliations:** 1 Department of Physics, National Chung Hsing University, 145, Xingda Rd., Taichung, 402 Taiwan, ROC; 2 Institute of Nanoscience, National Chung Hsing University, 145, Xingda Rd., Taichung, 402 Taiwan, ROC

**Keywords:** Integrated optics, Integrated optics, Mid-infrared photonics, Mid-infrared photonics

## Abstract

This paper presents an ultracompact tunable device for power splitting and switching by tuning the Fermi energy level of monolayer patternless graphene underneath a slotted multimode interference (MMI) coupler operating in the mid-infrared, λ = 9–11 μm. By introducing a high-index silicon slot in the central region of the MMI structure, which can significantly shorten the beat length, the proposed device has an approximately 4.5-fold reduction in device length and a two-fold improvement in power transmission compared with conventional MMI couplers without slotting. The device has a footprint of only 0.30 × 0.65 μm^2^ (<λ/10), making it the smallest power splitter and switcher. Over the bandwidth of 2 μm, the power transmission of the proposed device is nearly uniform. Extending the operating bandwidth is limited only by the practically achievable Fermi energy of graphene. For the fabrication tolerance, the numerical results show that the relative power variations are lower than 5%, even though the dimension variations are greater than 15%. With its advantages of tunability, compact footprint, and broadband operation, the proposed device is suitable for highly dense photonic integrated circuits.

## Introduction

To achieve high data transmission rates in optical networks or to build on-chip photonic integrated circuits, various optical devices, including power, wavelength, and polarization splitters, play pivotal roles in manipulating optical signals^1–3^. In addition to the data rate, it is essential to shrink the footprint of photonic devices to realize all-optical networks. In recent years, surface plasmon polaritons (SPPs) have been demonstrated to be a potential solution for reaching subwavelength-scale devices in the visible and near-infrared (near-IR) bands because of their ability to squeeze electromagnetic waves beyond the diffraction limit^4,5^. In principle, SPP guided modes are formed at metal–dielectric interfaces, where most of the electric field is perpendicular to the metal surface. Noble metals, including gold and silver, are often used to build plasmonic devices operating in the visible and near-IR bands. However, the SPP modes formed by the noble metals have poor mode confinement in the mid-IR regime^6,7^, restricting the device size and the on-chip density of integration. Therefore, alternative materials are needed for plasmonic devices operating in this regime. Graphene^8^, an emerging two-dimensional material with atomically thin sheets, has been numerically and experimentally demonstrated to support extremely confined and low-loss SPP modes in the terahertz and mid-IR frequencies^9–13^. Additionally, the optical properties of graphene sheets can be actively tuned by varying the Fermi energy level through electrostatic gating or chemical doping, not possible with conventional noble metals^7,14^. Thus, many graphene-based photonic devices operating in the mid-IR have been reported^15–19^. In contrast, graphene-based optical devices operating in the visible and near-IR bands suffer from substantial insertion loss; however, this can be moderately alleviated by deliberately designing a non-resonant metamaterial-based architecture^20^. In this study, we aim to design optical devices operating in the mid-IR regime.

Multimode interference (MMI) couplers are waveguide structures widely used in various optical devices, including power, wavelength, and polarization splitters, switchers, and add-drop multiplexers^21–26^. The principle of MMI coupling is based on self-imaging theory^27^, in which a property of an input field can be reproduced, according to the intermodal interference, in single or multiple images at periodic intervals along a multimode waveguide. Conventional MMI-based devices work well only at a designed central wavelength or within a narrow bandwidth^21–26^. For different wavelengths and also to control the bar-, cross-, or 3-dB image, one needs to modify the dimensions of the MMI-based devices. To overcome this, some researchers have applied the tunability of graphene to the design of MMI couplers for broadband applications with fixed device dimensions operating in the mid-IR^28–30^. Nonetheless, conventional MMI-based devices are still relatively long, hindering the realization of high-density photonic integrated circuits. To reduce the length of MMI-based devices, the shape of the MMI region can be changed^31,32^ or the number of excited modes can be restricted^33^. These approaches require either complicated (parabolically tapered) geometry^31,32^ or control of the power ratios of the excited modes^33^, limiting their usefulness. Recently, Mackie and Lee^34^ proposed a novel and simple modification. They introduced slots into the MMI region to reduce the self-imaging length by a factor of *N* + 1 for *N* slots, where the term “slot” refers to a narrow subregion of the MMI region.

In this paper, we combine the advantages of a slotted MMI coupler and a tunable graphene sheet to achieve a significant reduction in the length of a conventional MMI coupler. Unlike the work of Mackie and Lee^34^ in which the refractive index of the slot region was slightly lower than that of the MMI region, in this paper, we consider a high-contrast refractive index between the slot and MMI regions to shrink the device significantly. In particular, the picture used to explain the reduction factor of the self-imaging length fails when the refractive index of the slot is greater than that of the MMI region. Therefore, the conclusions of Mackie and Lee^34^ do not apply in this study. The operating band of the proposed device is in the mid-IR range between wavelengths λ = 9.0 and 11.0 μm. We also compared the length of the proposed device with a conventional MMI coupler without slots.

## Results

### Mode analysis of the proposed structure

**Figure 1:**
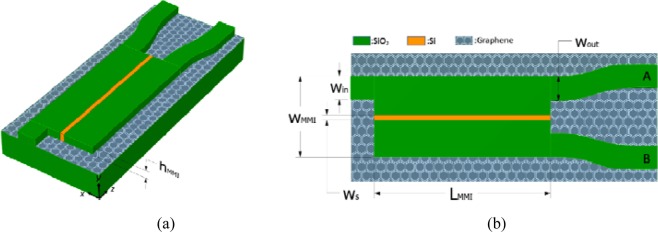
(**a**) 3D schematic diagram and (**b**) top view of the proposed graphene plasmonic slotted MMI coupler.

First, we numerically analyzed the mode properties of the proposed design. Figure 1(a,b) show a 3D schematic diagram and the top view of the device, respectively. The proposed structure consists of an MMI coupler made of SiO_2_. The central subregion has been replaced by an Si slot along its entire length, which is formed on top of a monolayer of patternless graphene, which has an atomically thin thickness of t_g_ on the underlying SiO_2_ substrate.

An input port of width W_in_ and two S-shaped bends of width W_out_ and radius of curvature R are connected at the entrance and exit of the MMI coupler, respectively. The thickness of the proposed structure is h_MMI_ for all parts and the length of the MMI coupler is L_MMI_. The widths of the input port, output port, slot, and the MMI coupler are W_in_, W_out_, W_S_, and W_MMI_, respectively. The relative permittivities of the Si and SiO_2_ used are *ε*_*Si*_ = 11.63 and *ε*_*SiO*2_ = 3.92^35^, respectively, for operating wavelengths between *λ* = 9.0 and 11.0 μm. The graphene is modeled as an anisotropic material with a thickness of t_g_ = 0.5 nm, and the relative permittivities along the out-of-plane and in-plane are *ε*_⊥_ = 2.5 and *ε*_*||*_ = 2.5 + *iσ*(ω)/ω*ε*_0_t_g_, respectively, where *σ*(ω) is the optical conductivity of graphene, calculated by the local random-phase approximation^36^ (as defined in the Method Section below). To produce an electrically controlled gating, the substrate needs to be deposited on a metal film. For a sufficient thickness of the SiO_2_ substrate (>300 nm for *E*_*F*_ < 1.1 eV), the back-gated metal film (not shown in Fig. 1(a)) does not affect the simulation results here.

**Figure 2:**
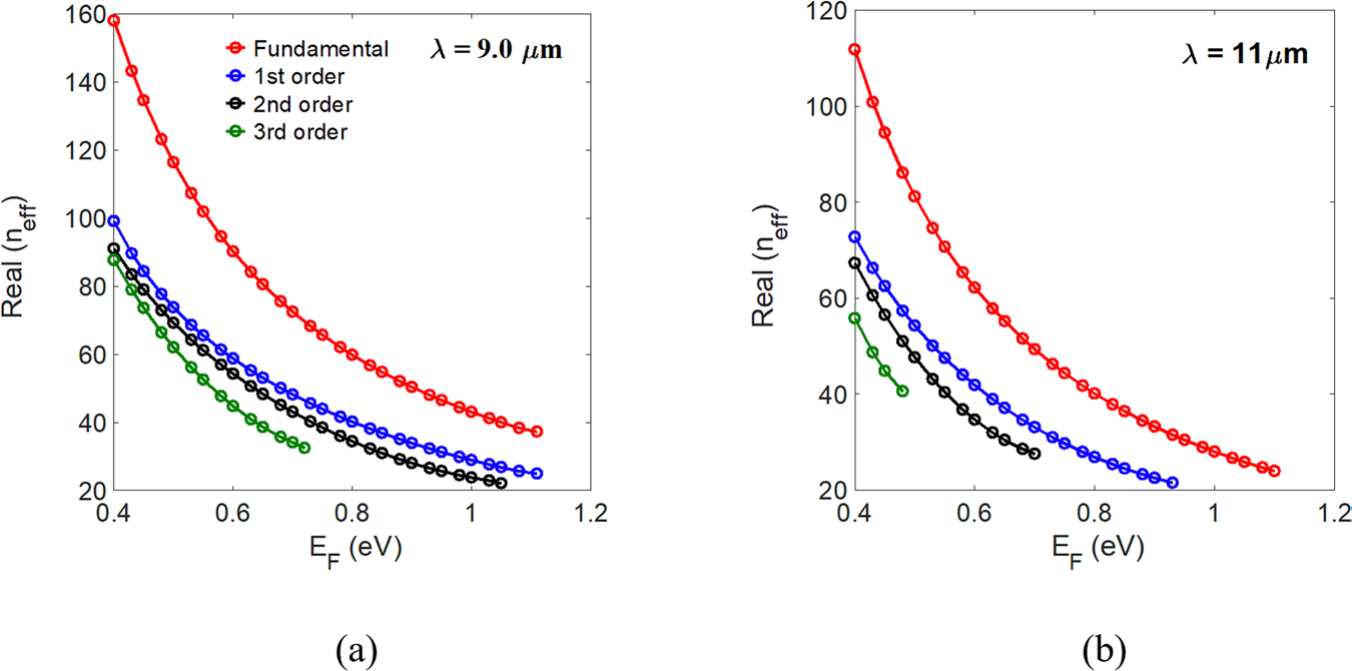
Real parts of the effective index of the four lowest-order modes of the MMI coupler versus the Fermi energy E_F_ of graphene at wavelengths (**a**) λ = 9.0 and (**b**) λ = 11.0 μm.

We consider the general interference (independence of the modal excitation and input location) from self-imaging theory^27^ to study the properties of the proposed device. A further reduction in its length can be achieved by considering the restricted interference by choosing the positions of input lights. According to general interference, single images of the input field can be reproduced at periodic intervals L_1_ = *p*(3L_π_) with *p* = 0, 1, 2, …, where *p* is an even or odd number for a direct or a mirrored replica (mirrored regarding the axis *x* = 0, as shown in Fig. 1(a), respectively, and L_π_ = *λ*/[2(*n*_eff,0_ − *n*_eff,1_)] is the beat length of the two lowest-order modes of the MMI structure. Here *n*_eff,0_ and *n*_eff,1_ are the effective indices of the fundamental and first-order modes, respectively. In addition to the single images, multiple images can be found at distances L_2_ = (*p*/2)(3L_π_) with *p* = 1, 3, 5, between the direct and mirrored imaging positions. We choose the smallest value, *p* = 1, when building a compact device. Due to the tunability of graphene, we can design broadband MMI-based devices with a fixed length. By introducing a high-index Si slot (unlike^34^, adopting a lower refractive index than that of the MMI region) into the central region of the MMI coupler, we can significantly increase the effective index of the symmetric fundamental mode while preserving the effective index of the antisymmetric first-order mode. Therefore, the difference in the effective indices of the two lowest-order modes can be significantly increased to shrink the length of the MMI device. Hence, we combine the advantages of a slotted MMI coupler and tunable graphene to realize a tunable submicron-scale power splitter and switcher. To the best of our knowledge, no other tunable slotted MMI-based devices have been reported so far. A direct (mirrored) replica appears at a distance L_MMI_ = 2L_π_ (3L_π_)^27^ and the width of the MMI coupler supports two (three) guided modes. The type of replica can be produced by tuning the E_F_ of graphene. We use the boundary-mode analysis of the commercial COMSOL Multiphysics software based on a rigorous finite element method. The computational window is surrounded by perfectly matched layers that absorb the outgoing light power effectively. The real parts of the effective index of the first four modes of the proposed device with W_MMI_ = 300 nm, h_MMI_ = 50 nm, and W_S_ = 30 nm versus the Fermi energy at wavelengths *λ* = 9.0 and 11.0 μm are shown in Fig. 2(a,b), respectively. The mesh size near the graphene sheet along the *y*-direction is set down to 0.1 nm to ensure sufficient precision.

**Figure 3:**
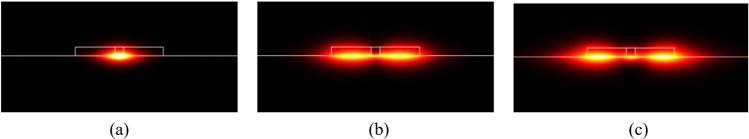
Magnitude of the electric fields |E| of the (**a**) fundamental, (**b**) first-order, and (**c**) second-order modes at the cross-section of the proposed slotted MMI coupler, having a width of W_MMI_ = 300 nm at E_F_ = 0.76 eV for a wavelength *λ* = 9.0 μm.

For *λ* = 9.0 μm, the proposed structure supports two (three) guided modes when E_F_ > 1.04 eV (0.72 eV). Therefore, to obtain the output power going through the upper (lower) branch (i.e., port A (B) in Fig. 1b), we choose the Fermi energy E_F_ = 1.05 eV (0.76 eV) to ensure the condition is satisfied. To obtain a 3-dB power splitter, E_F_ must be in the range 0.76 to 1.05 eV. For *λ* = 11.0 μm, tuning the Fermi energy to E_F_ = 0.70 eV (0.51 eV) obtains the output power going through the upper (lower) branch, as shown in Fig. 2(b). In the mode analysis, the Fermi energies of the graphene used are within 0.51 eV ≤ E_F_ ≤ 1.05 eV, the experimentally achievable range^16^. To show the mode profiles clearly, the magnitude of the electric fields |E| of the fundamental, first-order, and second-order modes for E_F_ = 0.76 eV at a wavelength *λ* = 9.0 μm are shown in Fig. 3(a–c), respectively. The effective indices are *n*_eff,0_ = 64.41–0.214*i* (*L*_*p*_ = 3.34 μm), *n*_eff,1_ = 43.12–0.136*i* (*L*_*p*_ = 5.27 μm), and *n*_eff,2_ = 37.60–0.144*i* (*L*_*p*_ = 4.97 μm), respectively, for the fundamental, first-order, and second-order modes, where *L*_*p*_ = 1/{2 Im(*k*^sp^)} is the propagation length and *k*^sp^ is the wave number of the guided SPP modes.

**Figure 4:**
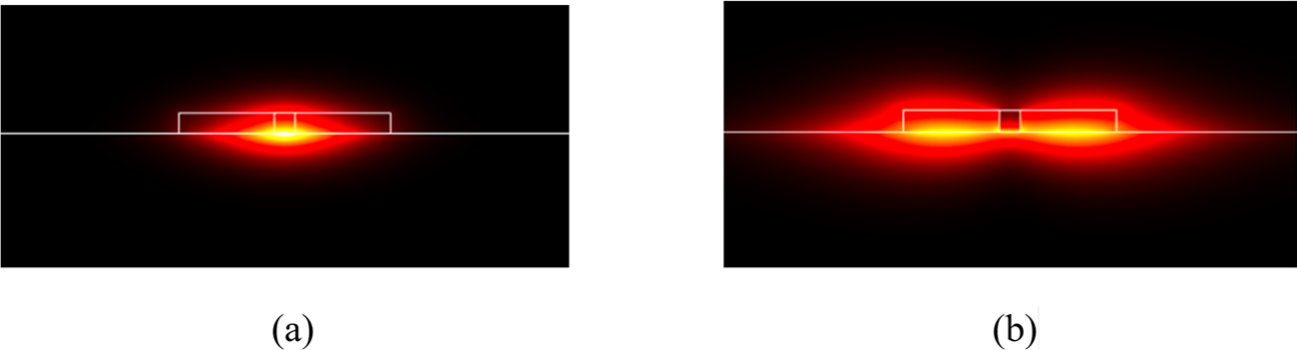
Magnitude of the electric fields |E| of the (**a**) fundamental and (**b**) first-order modes at the cross-section of the proposed slotted MMI coupler, having a width of W_MMI_ = 300 nm at E_F_ = 1.05 eV for a wavelength *λ* = 9.0 μm.

Similarly, the magnitude of the electric fields |E| of the fundamental mode (*n*_eff,0_ = 39.99–0.080*i*; *L*_*p*_ = 8.95 μm) and first-order mode (*n*_eff,1_ = 26.81–0.055*i*; *L*_*p*_ = 13.02 μm) for E_F_ = 1.05 eV at the wavelength *λ* = 9.0 μm are shown in Fig. 4(a,b), respectively.

**Figure 5:**
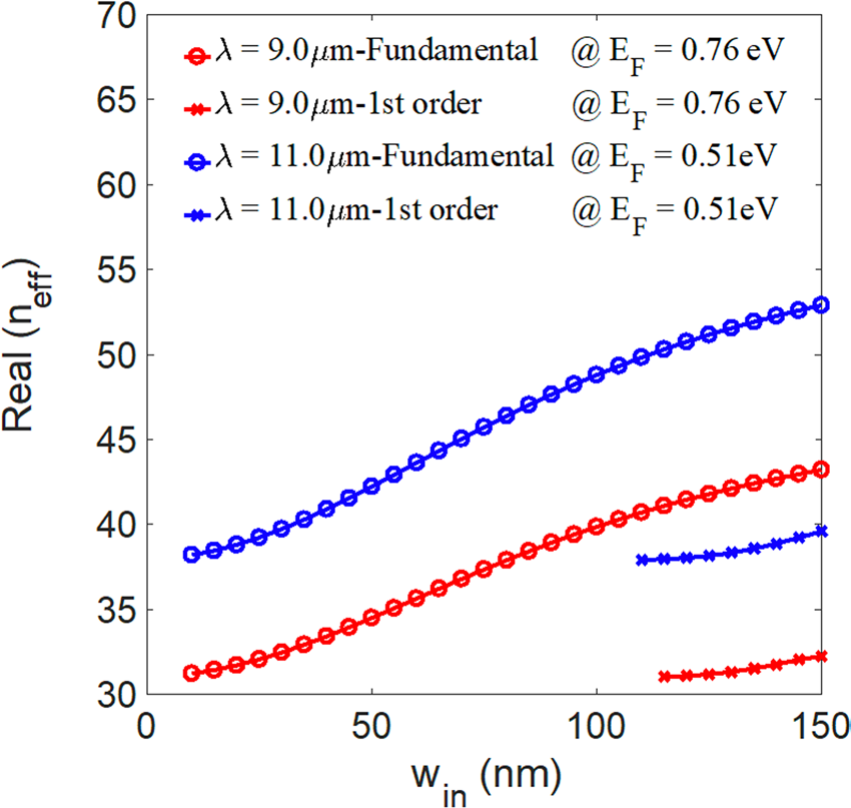
Real parts of the effective index of the fundamental and first-order modes versus the width of the input port at the Fermi energies E_F_ = 0.76 and 0.51 eV for the wavelengths *λ* = 9.0 and 11.0 μm, respectively.

As Figs 3 and 4 show, we observe that the mode confinement and energy loss decrease as the Fermi energy of the graphene increases. We also investigate the mode characteristics of the input waveguides to ensure we have single-mode operation (here, we assume W_in_ = W_out_). The real parts of the effective index versus the width W_in_ at the Fermi energies E_F_ = 0.76 (the smallest Fermi level for *λ* = 9.0 μm) and 0.51 eV (the smallest Fermi level for *λ* = 11.0 μm) for the wavelengths *λ* = 9.0 and 11.0 μm, respectively, are shown in Fig. 5. The numerical results show that the width of the input port must satisfy the conditions W_in_ < 110 nm and W_in_ < 105 nm for the wavelengths λ = 9.0 and 11.0 μm, respectively, to preserve the single-mode operation. In this work, we chose the width of the input port as W_in_ = 100 nm in the subsequent analysis.

### Performance and fabrication tolerance of the proposed structure

To study the propagation properties, we performed 3D simulations to study the power transmission and operating bandwidth. The two S-shaped output waveguides consist of two connected circular arc waveguides with opposite curvature. To circumvent the bending loss, we used a radius of curvature of 1.5 μm over 0.28 radians in this work. Once the fundamental mode of the input waveguide is launched into the proposed MMI structure, it excites the supportable number of modes. For the wavelength *λ* = 9.0 μm, the difference between the effective indices of the fundamental and first-order modes is Δ*n* = Re(*n*_eff,0_ − *n*_eff,1_) = 21.29 for the Fermi energy E_F_ = 0.76 eV supporting three guided modes, as shown in Fig. 2(a). Hence, to form a mirrored image (at the lower branch, B), the length of the proposed device is L_MMI_ = L_1_ = 3L_π_ = 635 nm. To form a direct image, we obtain L_MMI_ = 683 nm for E_F_ = 1.05 eV. To optimize the imaging quality by balancing the phase errors, a slight adjustment of the imaging distance is required because the predictions from the self-imaging theory are approximations. By tuning the Fermi energy to E_F_ = 0.93 eV, the proposed device behaves as a two-way power splitter. Therefore, we choose the length of the proposed design as L_MMI_ = 650 nm for the subsequent calculations.

**Figure 6:**
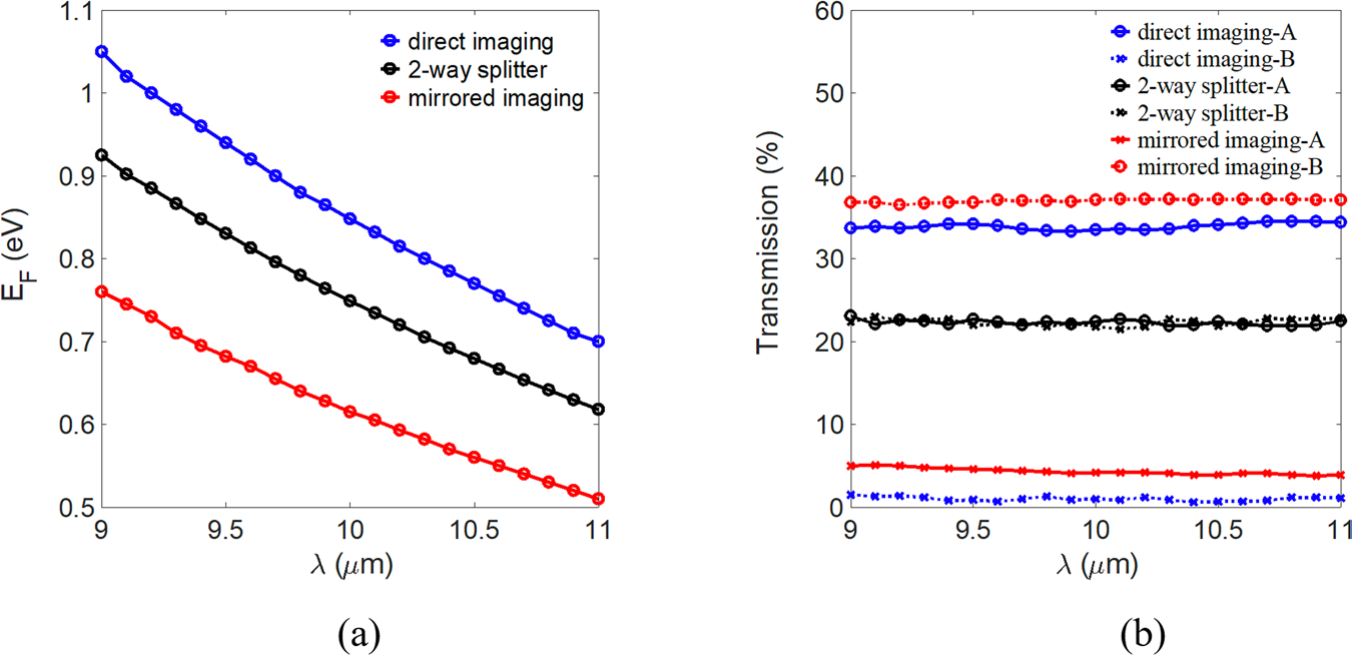
(**a**) E_F_ and (**b**) transmission versus the operating wavelength λ.

Figure 6(a,b) show the Fermi energy and output power transmission, respectively, versus the operating wavelength from *λ* = 9.0 to 11.0 μm for the three imaging conditions. We calculated the power transmission at the location of 150 nm from the end of the MMI coupler. For all imaging conditions, E_F_ decreases as *λ* increases, as shown in Fig. 6(a), because a lower Fermi energy results in stronger mode confinement.

The calculated transmission is approximately 35% (37%) at port A and 2% (4%) at port B for the direct (mirrored) imaging condition. For the two-way splitter, we obtain the same transmission of approximately 22% at both ports. The proposed device has stable power transmission over the entire operating bandwidth, as shown in Fig. 6(b). In Table 1, we list the power transmissions and Fermi levels of the three imaging conditions under different wavelengths to discuss their advantages and limitations. We find that the extinction ratios (~15 dB) of the direct imaging are higher than those (~10 dB) of the mirrored imaging. However, the transmissions (~34%) of the direct imaging are slightly lower.

**Table 1:** Power transmission (%) of the three imaging conditions under different wavelengths.

Imaging condition	Direct			Mirrored			2-way splitter		
Wavelength	P_A_	P_B_	E_F_ (eV)	P_A_	P_B_	E_F_ (eV)	P_A_	P_B_	E_F_ (eV)
λ = 9 μm	33.7	1.5	1.05	36.8	5.0	0.76	23.1	22.4	0.93
λ = 10 μm	33.5	1.0	0.85	37.2	4.2	0.61	22.4	21.8	0.75
λ = 11 μm	34.4	1.1	0.70	37.1	3.9	0.51	22.5	22.8	0.62

**Figure 7:**
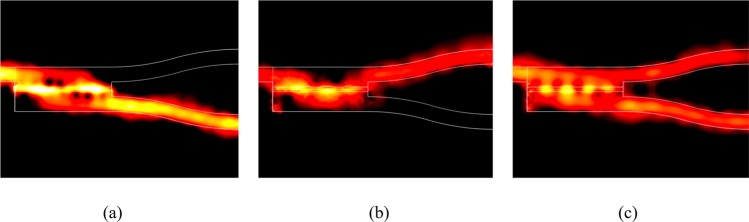
Electric field profiles |E| of (**a**) the mirrored image, (**b**) the direct image, and (**c**) the two-way splitter for Fermi energy levels E_F_ = 0.62, 0.85, and 0.75 eV, respectively, for a wavelength λ = 10.0 μm.

Both of the extinction ratio and transmission are wavelength-insensitive over the bandwidth of 2 μm. For the 2-way splitter, the imbalance (i. e., IB = 10log10(P_A_/P_B_)^37^) is smaller than 0.14 dB. In addition, the direct imaging of the proposed structure cannot be obtained for shorter wavelength (<9 μm) if the achievable Fermi level of graphene is lower than 1.1 eV^16^. Note that the power transmission can be improved by using tapered waveguides to reduce the reflections at the connection points between the MMI structure and the input or output waveguide. Additionally, extending the operating wavelength is restricted only by the practically accessible range of the Fermi energy. To observe the light propagation, the electric field profiles |E| at the top of the graphene sheet for the mirrored, direct, and two-way splitter imaging conditions for the wavelength *λ* = 10.0 μm are shown in Fig. 7. The white lines indicate the profile of the proposed device.

**Figure 8:**
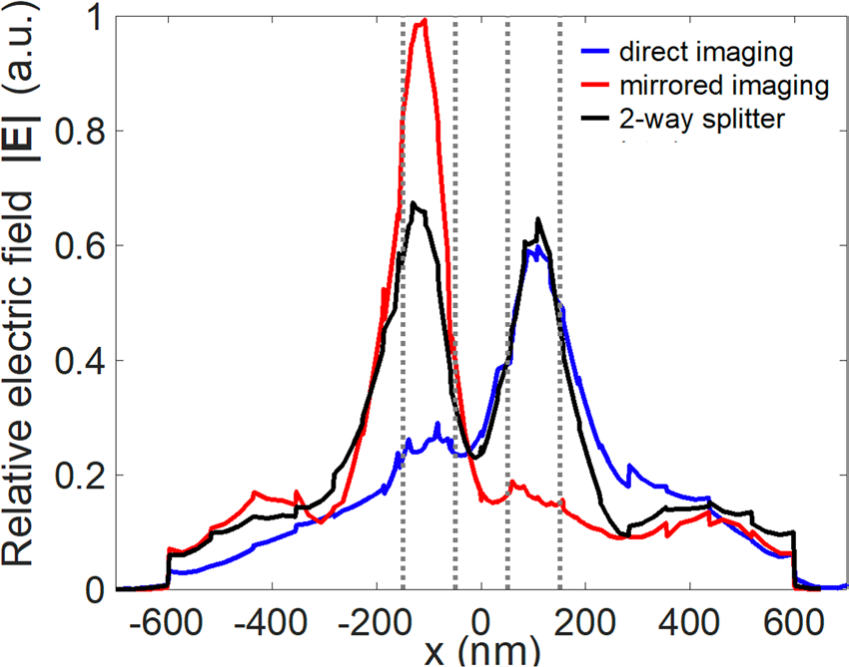
Relative field amplitude |E| along the *x*-direction for the three imaging conditions at the location of 150 nm from the end of the MMI structure for the wavelength λ = 10.0 μm. The gray dotted lines indicate the boundaries of the output ports.

These results clearly demonstrate that power switching and splitting can be realized by the proposed slotted MMI-based structure, with only 300 nm wide and 650 nm long, by varying the Fermi energy of graphene. The relative field profiles along the *x*-direction of the three conditions are shown in Fig. 8.

We observed that there are some haphazard peaks of the relative field amplitude along the *x*-direction for the three imaging conditions. Theoretically, the phases of the different guided modes will accumulate along the propagation distances with different wave numbers in the process of forming image. Therefore, small deviations from the calculated phases of the beat length (an approximation) lead to blur the reconstructed image field. As a result, this phenomenon can be explained by the imaging quality, which is defined by how accurately the input field is reproduced at the imaging position of a multimode waveguide. Roughly, the definition of the imaging resolution is proportional to the effective width of the highest supported mode profile divided by the mode number^27^. Therefore, the mirrored image, composed of three guided modes, has a better imaging quality than the direct image composed of two guided modes. Obviously, the mirrored image has a higher field amplitude, stronger mode confinement, and lower ripple than the direct image. In addition, the device length is also moderately adjusted to be 650 nm making the haphazard peaks of the relative field amplitude heavier. Therefore, the mirrored image, composed of three guided modes, has a better imaging quality than the direct image composed of two guided modes. Obviously, the mirrored image has a higher field amplitude, stronger mode confinement, and lower ripple than the direct image.

**Figure 9:**
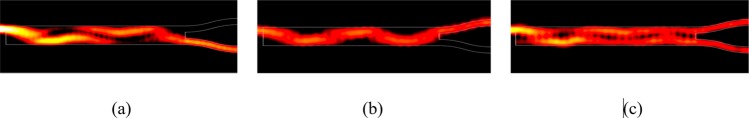
Field flow profiles |E| of (**a**) the mirrored image, (**b**) the direct image, and (**c**) the two-way splitter for Fermi energy levels E_F_ = 0.51, 0.76, and 0.63 eV, respectively, for a conventional MMI coupler without a slot for a wavelength λ = 10.0 μm.

For a conventional MMI coupler without slots, the difference between the effective indices of the fundamental and first-order modes is only Δ*n* = Re(*n*_eff,0_ − *n*_eff,1_) = 5.0 (6.4) at a wavelength of *λ* = 10.0 μm for a Fermi energy E_F_ = 0.51 eV (0.76 eV) for the mirrored (direct) image. Hence, the device is approximately 2900 nm long after adjusting for the phase errors from the self-imaging theory^27^. A two-way splitter can be obtained by tuning the Fermi energy to E_F_ = 0.63 eV, between that for the mirrored and direct imaging conditions. The electric field profiles |E| of the mirrored image, direct image, and two-way splitter are shown in Fig. 9(a–c), respectively. For the mirrored (direct) imaging condition, the calculated power transmission at the same location as that of the proposed design is approximately 15% (18%) at port A and 1% (2%) at port B. It is approximately 14% at the two ports for the two-way splitter.

**Figure 10:**
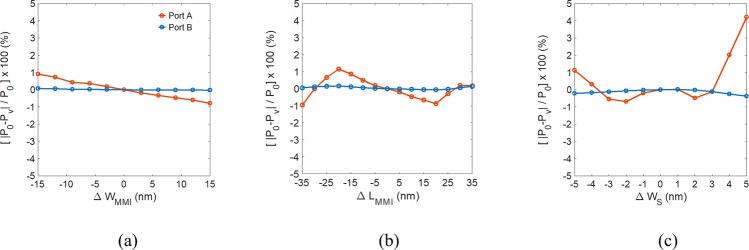
Relative power variation versus variations of (**a**) W_MMI_, ΔW_MMI_, (**b**) L_MMI_, ΔL_MMI_, and (**c**) W_S_, ΔW_S_ of the proposed structure.

The results show that the proposed design has an approximately two-fold improvement in transmission because of the absorption loss due to the metal plasmon of the longer graphene sheet, 4.5 times longer than that of the proposed design. In addition to the dimensions and performance, fabrication tolerance is also a critical issue when building robust devices. For a mirrored image at a wavelength *λ* = 10.0 μm, the relative power variation, defined by [|*P*_0_ - *P*_v_|/*P*_0_] × 100%, versus the geometrical errors in W_MMI_, L_MMI_, and W_S_ is shown in Fig. 10(a–c), respectively, where *P*_0_ and *P*_v_ are the power transmission for the original and varied dimensions.

The relative power variations of port B versus ΔW_MMI_ (±15 nm), ΔL_MMI_ (±35 nm), and ΔW_S_ (±5 nm) are far smaller than 1% because the image (major power) is formed in port B. For port A with minor power, the relative power variations versus ΔW_MMI_ and ΔL_MMI_ are approximately 1%. The relative power variation versus ΔW_S_ is still smaller than 5% when ΔW_S_ =  ± 5 nm. These results confirm that the performance of the proposed structure is robust regarding fabrication errors. The most critical parameter is the width of the silicon slot W_S_. Fortunately, the dimensions of the Si slot can be controlled to be precisely under 10 nm by modern fabrication techniques, such as low-pressure chemical vapor deposition^38^.

In conclusion, we have proposed an ultracompact tunable device operating in the mid-IR between λ = 9 and 11 μm. It is based on a slotted MMI coupler deposited onto a graphene sheet. By varying the Fermi energy level of the graphene using electrostatic gating, the proposed device can act as a power splitter or as a switcher. By introducing a high-index Si slot into the central region of the MMI coupler, the footprint of the proposed design is only at the submicron scale, 0.30 μm × 0.65 μm. To the best of our knowledge, this is the shortest device operating in the mid-IR. Compared with a conventional MMI coupler without a slot, our reported device has an approximately 4.5-fold reduction in device length and a two-fold improvement in power transmission. Moreover, the operating bandwidth is restricted only by the practically achievable Fermi level. For the fabrication tolerance, the relative power variations are smaller than 5%, even when the geometrical deviations are greater than 15%. Further shrinking the device footprint is feasible by choosing or artificially designing materials with a higher refractive index for the slot region. Our work is a potential approach for significantly increasing the density of integration of photonic integrated circuits.

## Methods

The optical conductivity of graphene, *σ*(ω), is calculated by the local random-phase approximation^36^:

1 $$\begin{array}{ccc}\sigma (\omega ) & = & \frac{i2{e}^{2}{k}_{B}T}{\pi {\hslash }^{2}(\omega +i{\tau }^{-1})}\,\mathrm{ln}[2\,\cosh (\frac{{E}_{F}}{2{k}_{B}T})]\\  &  & +\,\frac{{e}^{2}}{4\hslash }\,\mathrm{ln}[\frac{1}{2}+\frac{1}{\pi }{\tan }^{-1}(\frac{\hslash \omega -2{E}_{F}}{2{k}_{B}T})-\frac{i}{2\pi }\,\mathrm{ln}(\frac{{(\hslash \omega +2{E}_{F})}^{2}}{{(\hslash \omega +2{E}_{F})}^{2}+{(2{k}_{B}T)}^{2}})].\end{array}$$

where *e* is the electron charge, *k*_B_ is the Boltzmann constant, *T* is the temperature, *ω* is the angular frequency of the incident light, *ћ* is the reduced Plank constant, E_F_ is the Fermi energy level, and *τ* = *μ*E_F_*/eV*_*F*_^*2*^ is the carrier relaxation lifetime. *μ* is the carrier mobility in graphene and *V*_*F*_ = 10^6^ m/s is the Fermi velocity of electrons. The Fermi energy E_F_ can be tuned by electrostatic gating. In recent experiments, the carrier mobility ranged from >4 m^2^ V^−1^ s^−1^ ^39,40^ in graphene grown by chemical vapor deposition to >20 m^2^ V^−1^ s^−1^ ^41^ in suspended exfoliated graphene. In this work, we adopt the reasonable value of *μ* = 10 m^2^ V^−1^ s^−1^ to calculate the carrier relaxation lifetime.
